# Detection of S-Nitrosothiol and Nitrosylated Proteins in *Arachis hypogaea* Functional Nodule: Response of the Nitrogen Fixing Symbiont

**DOI:** 10.1371/journal.pone.0045526

**Published:** 2012-09-19

**Authors:** Debasis Maiti, Tuhin Subhra Sarkar, Sanjay Ghosh

**Affiliations:** Department of Biochemistry, University of Calcutta, Kolkata, West Bengal, India; Laurentian University, Canada

## Abstract

To detect the presence of NO, ROS and RNS in nodules of crack entry legumes, we used *Arachis hypogaea* functional nodule. The response of two cognate partner rhizobia was compared towards NO and GSNO using *S. meliloti* and *Bradyrhizobium sp* NC921001. ROS, NO, nitrosothiol and bacteroids were detected by fluorescence microscopy. Redox enzymes and thiol pools were detected biochemically. Nitrosothiols were found to be present but ROS and NO were absent in *A. hypogaea* nodule. A number of S-nitrosylated proteins were also detected. The total thiol pool and most of the redox enzymes were low in nodule cytosolic extract but these were found to be high in the partner microorganisms indicating partner rhizobia could protect the nodule environment against the nitrosothiols. Both *S. meliloti* and *Bradyrhizobium sp* NC921001 were found to contain GSNO reductase. Interestingly, there was a marked difference in growth pattern between *S. meliloti* and *Bradyrhizobium sp* in presence of sodium nitroprusside (SNP) and S-nitrosoglutathione (GSNO). *Bradyrhizobium sp* was found to be much more tolerant to NO donor compounds than the *S. meliloti*. In contrast, *S. meliloti* showed resistance to GSNO but was sensitive to SNP. Together our data indicate that nodule environment of crack entry legumes is different than the nodules of infection mode entry in terms of NO, ROS and RN*S.* Based on our biochemical characterization, we propose that exchange of redox molecules and reactive chemical species is possible between the bacteroid and nodule compartment.

## Introduction

Nitric oxide (NO) is a small lipophilic molecule with immense physiological and pathological roles in biological system [1]. NO is involved in a number of physiological processes in plants [2], [3]. Presence of nitric oxide synthase activity was shown in roots and nodules of *Lupinus albus* [4]. Despite the completion of a plethora of plant genome sequences, genes encoding the NO synthesizing enzymes known as nitric oxide synthases (EC 1.14.13.39) have not been identified in plants. NO can be produced by other sources, such as xanthine oxidoreductase, peroxidase and cytochrome P450 [5]. Although descriptions of NO-mediated processes are accumulating, the plant signaling pathways governed by NO are still largely unknown [6], [7]. NO regulates the activity of numerous proteins by S- or metal nitrosylation and tyrosine nitration. In plant systems, S-nitrosylation of proteins are found to contribute to gene regulation [8]–[11], modulates phytohormonal signaling [12] and cell death [13]–[15]. Recently, glutamine synthetase has been found to be a molecular target of nitric oxide in root nodules of *Medicago truncatula* where it is regulated by tyrosine nitration [16]. However, the biological significance of protein nitration is still not clearly understood.

*Arachis hypogaea* (peanut or groundnut), a well-known member of the Leguminosae family, subfamily Papilionoideae, tribe Aeschynomeneae is an important oil seed crop. The interaction between legume plants and some soil bacteria, collectively called rhizobia, leads to the establishment of symbiotic relationships, characterized by the formation of new differentiated root organs called nodules which provide a niche for bacterial nitrogen fixation. *Arachis* genus consists of 22 species among which 9 species are reported to be nodulated [17], [18]. The distinctive features of aeschynomenoid nodules have been studied in depth [19]–[22]. *Arachis* roots are distinctly different from other root systems in lacking root hairs. However, tufted clusters or rosettes of multicellular hairs are found in young roots in the junction of the taproot and lateral root of *Arachis* which are thought to be important for bacterial invasion [23]–[25]. The penetration occurs through epidermal breaches i.e. crack at sites where lateral roots protrude in *A. hypogaea* [26]. Nod factor has been identified from *Arachis-Bradyrhizobium sp* interaction [27] but it is not yet known whether *Arachis sp* like the other members of the aeschynomenoid/dalbergioid tribe, can undergo successful nodulation in the absence of nod factor [28]. Another mode of infection of legumes by rhizobia may occur through root hair invasion [29] as for e.g. in *M. trancatula* infected by *Sinorhizobium meliloti*. Transient presence of NO was observed in *Lotus japonicus* [30] and *Medicago sativa* [31] roots within a few hours after infection by their cognate symbiont. NO is found to be produced in infection threads and nodule primordia of *M. trancatula* [32]. NO bound to leghemoglobin (Lb) as nitrosyl hemoglobin (LbNO) was detected in nodules of soybean infected by *Bradyrhizobium japonicum* [33]. Moreover, Baudouin and colleagues clearly showed the presence of NO in functional nodules of *M. trancatula* infected by *S. meliloti* [34]. Pii and coworkers confirmed this observation and extended it to *M. sativa* [35]. Recently, it has been shown that NO is involved in the regeneration of energy in *M. truncatula* functioning nodules [36]. In contrast to all that has been carried out in nodules of legumes where infections occur through root hair invasion, the response of nitric oxide in nodules evolved through crack mode entry has been poorly documented. In addition, how rhizobia cope with the presence of NO in nodules and what role is played by the bacterial NO response in the interaction with the host plants are not clearly known.

To detect the presence of NO, ROS and RNS in nodules of crack entry legumes, we used *A. hypogaea* functional nodules. The response of rhizobia towards RNS was studied in *Bradyrhizobium sp* NC921001 and *S. meliloti*. In this study we report the presence of S-nitrosothiols, not nitric oxide in the functional *A. hypogaea* nodules. *Bradyrhizobium sp* was found to be much more tolerant to NO donor compounds than the *S. meliloti*. Our biochemical studies showed that the partner symbiont might have some contributions to restore the cellular redox environment against S-nitrosothiols. We also report the presence of S-nitrosylated proteins in functional *A. hypogaea* nodules suggesting importance of S-nitrosylation in NO dependent signal transduction in nodule environment.

## Results

### Absence of Free Nitric Oxide and ROS in *A. hypogaea* Nodules

**Figure 1:**
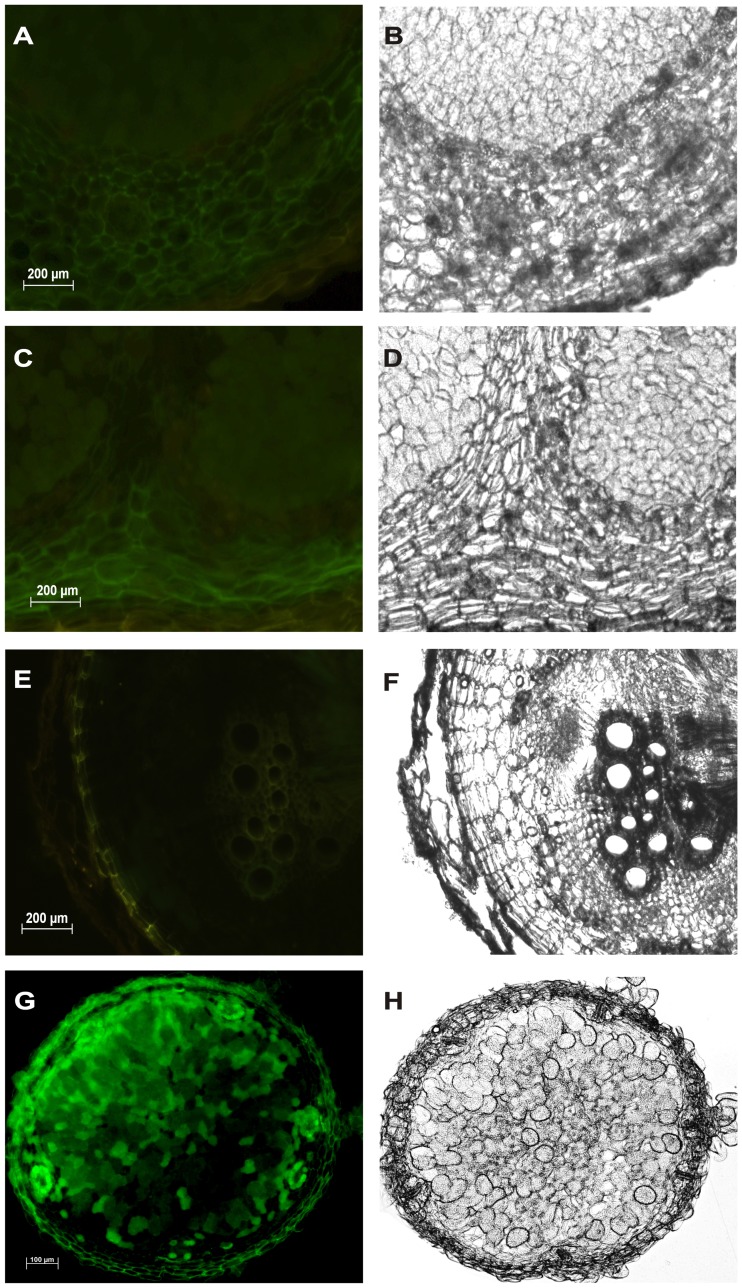
NO detection in *A. hypogaea* JL 24 and *M. sativa* nodules. All nodule sections were incubated with 10 µM DAF-2DA at 25°C for 1 hour in darkness. Photographs are representative of results obtained from the analysis of nodules in five independent experiments. Images of *A. hypogaea* nodule sections show absence of NO dependent DAF-2DA fluorescence (green colour) in (A) 20 day, (C) 40 day and (E) 80 day old nodules. (B), (D) and (F) show the corresponding bright field images. Scale bar = 200 µm. Whereas DAF-2DA dependent green colour fluorescence was observed in (G) 143 day old *M. sativa* nodule section. (H) represents the corresponding bright field image. Scale bar = 100 µm. The images of the sections were captured using excitation at 485 nm and emission at 530 nm.

The presence of nitric oxide has been reported in 10, 20 and 30 day old nodules of *M. trancatula* and 40 day old functional nodules of *M. sativa* using DAF-2DA by two different laboratories [34], [35]. To detect the presence of nitric oxide in *A. hypogaea* functional nodules of 20, 40 and 80 day, nodule sections were stained with cell permeable NO specific probe 4, 5-diaminofluorescein diacetate (DAF-2DA) [37]. *M. sativa* nodules of 143 day collected from agricultural firm were used as a positive control. *A. hypogaea* nodule sections did not show any NO specific fluorescence (Figure 1 Panel A, C and E). However, nodule sections of *M. sativa* showed significant positive NO specific fluorescence (Figure 1 Panel G) indicating that nitric oxide is formed in *M. sativa* nodules of different ages (40 day [35] and 143 day). This result was confirmed by treating nodule slices with the NO scavenger 2-(4-carboxyphenyl) - 4, 4, 5, 5-tetramethyl imidazoline-1-oxyl-3-oxide (cPTIO) (Figure S1). There was no ROS specific fluorescence in nodule sections of *A. hypogaea* (20, 40 and 80 day) using Dichlorofluorescein diacetate (DCF-DA). Nodule sections of *M. sativa* (143 day) also showed similar observations (Figure S2).

### Presence of Nitrosothiols and Bacteroids in *A. hypogaea* Nodules

**Figure 2:**
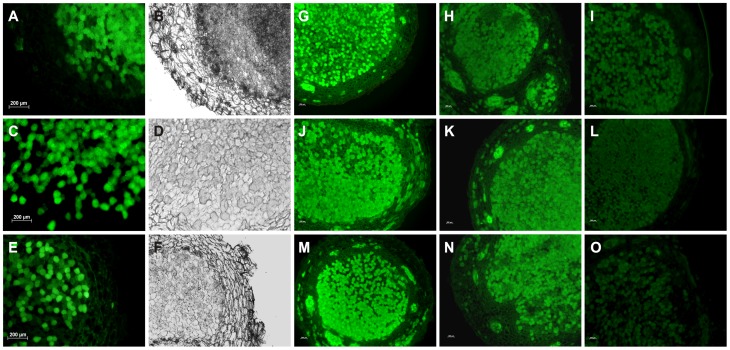
RSH and/or RSNO detection in *A. hypogaea* JL 24 nodules. *A. hypogaea* nodule sections were incubated with 10 µM Alexa fluor 488 Hg-linked phenylmercury at 25°C for 2 hour in darkness. Photographs are representative of results obtained from the analysis of nodules in five independent experiments. Images of nodules show RSH, RSNO, disulphide linkage and background dependent Alexa fluor 488 fluorescence (green colour) in (A) 20 day, (C) 40 day and (E) 80 day old nodules. (B), (D) and (F) show the corresponding bright field images. Further experiments were performed to confirm the presence of RSNO in the nodule sections. Control set cross sections of 20 (G), 40 (J) and 80 (M) day old nodules were treated with buffer only; corresponding sections of different day old nodules (H), (K), (N) were treated with 50 mM L-NEM and were incubated at 25°C for 2 h in darkness; sections (I), (L), (O) were incubated with 20 mM ascorbate for 1 hour at room temperature followed by similar NEM treatment. All the sections were stained with 10 µM alexafluor 488 at 25°C for 2 h in darkness. (G), (J), (M) depicts the presence of RSH, RSNO, disulphide linkage and background fluorescence; the fluorescence of (H), (K), (N) were due to RSNO, disulphide linkage and background fluorescence whereas (I), (L), (O) represents the fluorescence for disulphide linkage and back ground. The fluorescence intensity difference between (H, I), (K, L), and (N, O) confirms for the presence of RSNO (for calculation see further Figure 4) in the corresponding day of *A. hypogaea* nodule sections. Scale bar = 200 µm.

**Figure 3:**
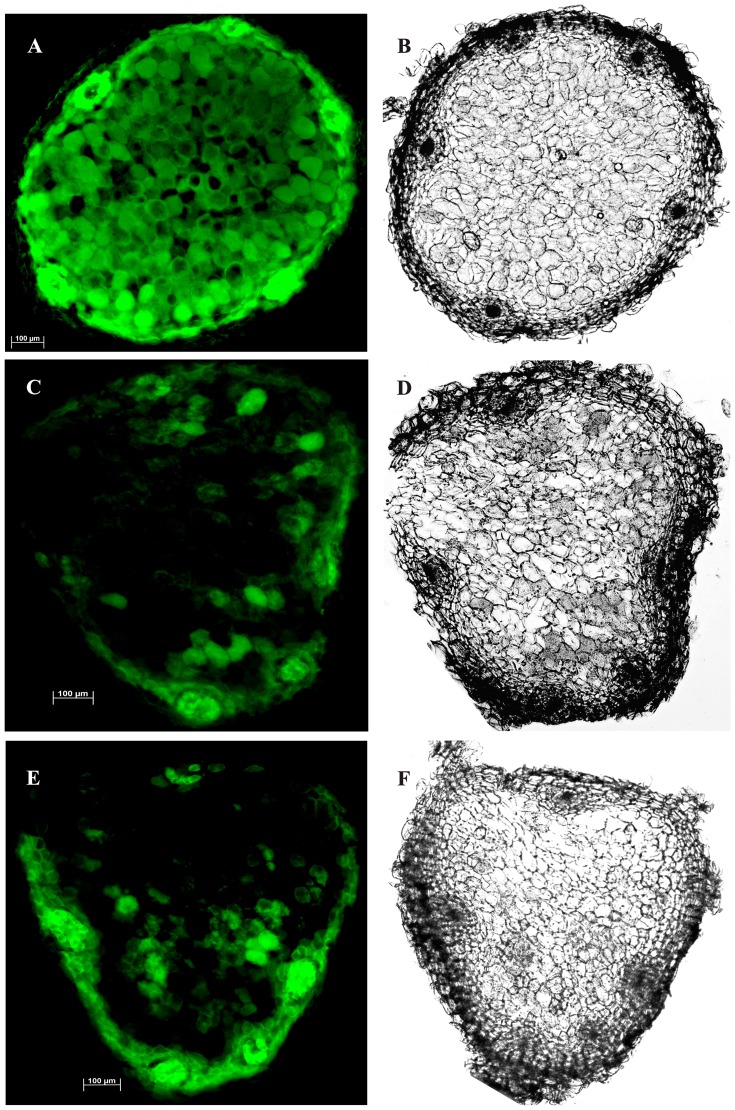
Detection of RSNO in *M. sativa* nodules. Control set cross sections of 143 day old *M. sativa* nodules (A) were treated with buffer only; sections (C) were treated with 50 mM L-NEM and were incubated at 25°C for 2 h in darkness; sections (E) were incubated with 20 mM ascorbate for 1 hour at room temperature followed by similar NEM treatment. All the sections were stained with 10 µM alexafluor 488 at 25°C for 2 h in darkness. Photographs are representative of results obtained from the analysis of nodules in five independent experiments. Green fluorescence of (A) depicts the presence of RSH, RSNO, disulphide linkage and background fluorescence, the fluorescence of (C) is due to RSNO, disulphide linkage and background fluorescence whereas (E) represents the fluorescence for disulphide linkage and back ground. The fluorescence intensity is not downward for E from C. This confirms the absence of RSNO (for calculations see further Figure 4) in the *M. sativa* nodule sections. (B), (D), and (F) represents the corresponding bright field images. Scale bar = 100 µm.

**Figure 4:**
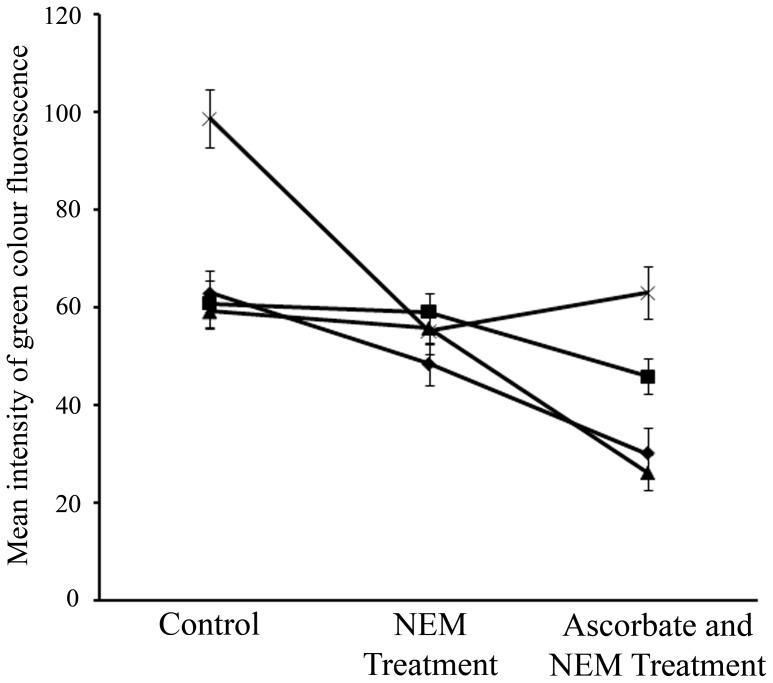
RSNO fluorescence intensity profile of nodules from *A. hypogaea* JL 24 and *M. sativa*. The mean intensity of green colour (as observed in Figure 2 and 3) of nodule sections was plotted for control, those were blocked with L-NEM and those were reduced with ascorbate and were blocked with L-NEM. This was done for both the *A. hypogaea* JL 24 (—♦— 20 day, —▪— 40 day and —▴— 80 day old nodule) and *M. sativa* (—×— 143 day old nodule). Results were expressed as mean ± SD, for n = 3 experiments.

Alexafluor 488 is Hg-link phenylmercury compound, which forms stable thiolates with free sulfhydryls as well as other thiol moieties including nitrosylated thiols [38]. Figure 2 and Figure 3 represent the presence of nitrosothiols in nodule sections of *A. hypogaea* and *M. sativa* stained with alexafluor 488. Presence of nitrosothiol was confirmed by using N-ethylmaleimide (L-NEM) as a thiol blocking agent. There was ∼23% decrease in fluorescence intensity in *A. hypogaea* 20 day old nodule sections following L-NEM addition (Figure 4) indicating free thiol content was low in it. The decrease in fluorescence intensity was found to be negligible in 40 day (∼2.8%) and 80 day old nodule sections (∼5.7%) of *A. hypogaea* following L-NEM treatment. A further decrease in fluorescence intensity was observed in 20 day (∼38%), 40 day (∼22%) and 80 day (∼53%) old nodule sections of *A. hypogaea* when they were reduced with ascorbate followed by blocking with L-NEM indicating the presence of significant amount of nitrosothiols. On the other hand, there was a ∼48% decrease in fluorescence intensity following the addition of L-NEM in nodule sections of *M. sativa* indicating high free thiol content present in it. The mean intensity of green color fluorescence was plotted to reproduce the intensity curve using Image J software (Figure 4). This figure clearly shows the differential fluorescence intensity profile of alexafluor 488 for thiols and nitrosothiols for both the plants’ nodules.

**Figure 5:**
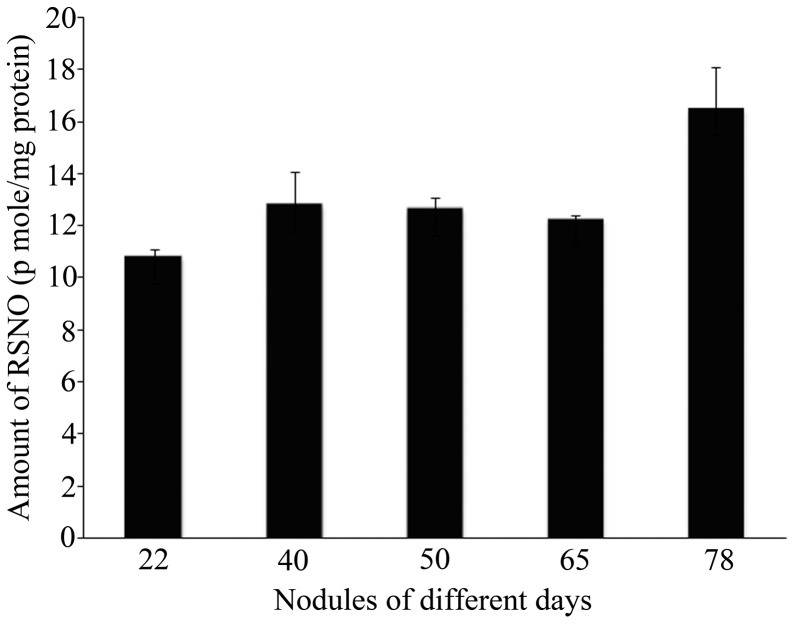
Biochemical quantification of Nitrosothiol by Saville assay. Quantification of nitrosothiol in 22, 40, 50, 65 and 78 day old nodule extracts of *A. hypogaea* was done by Saville assay. Results were expressed as mean ± SD, for n = 3 experiments. P≤0.01, using one-way ANOVA.

**Figure 6:**
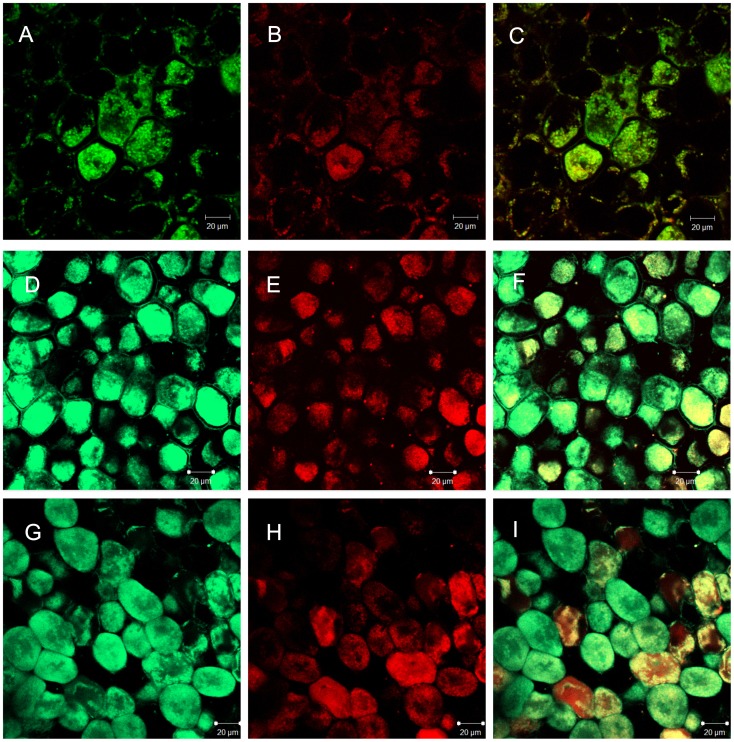
Colocalization of nitrosothiol (RSNO) and bacteroids in the *A. hypogaea* JL 24 nodules. 20 day (A, B, C), 40 day (D, E, F) and 80 day (G, H, I) old nodules cross sections were incubated in the presence of 10 µM Alexafluor 488 and 10 µM Syto83 at 25°C for 2 hour in darkness and fluorescence were observed under confocal laser microscopy. Photographs are representative of results obtained from the analysis of nodules in three independent experiments. The fluorescence associated with (A), (D), and (G) was for RSNO detection and (B), (E) and (H) for bacteroids. (C), (F) and (I) are the overlaying corresponding images (A and B, D and E, G and H respectively). Scale bar = 20 µm.

Saville assay is specific for quantification of nitrosothiols in terms of p mole nitrite mg^−1^ protein. Figure 5 represents nitrosothiol profile of 22, 40, 50, 65 and 78 day old nodule extracts of *A. hypogaea.* Nitrosothiol content was changed significantly in different crude nodule extracts of different time points ranging from 10.8±0.3 to 16.47±1.61 p mole nitrite mg^−1^ proteins. We used a fluorescent nucleic acid dye of the Syto family, which has been previously reported as being efficient for bacteroid detection in nodules [39]. In regard to the spectral characteristics of the different Syto molecules, we chose the Syto83 dye, the fluorescent emission of which can be fully discriminated from the alexafluor 488 fluorescent emission. Nodule slices were cotreated with Syto83 and alexafluor 488 dyes and were subsequently observed by confocal microscopy. As shown in Figure 6, a strong correlation between the cells that contained bacteroids and the cells that were producing nitrosothiol was observed. Thus it can be concluded that bacteroids are exposed to nitrosothiols within the nodule environment.

### Presence of Nitrosylated Proteins in Functional Nodules of *A. hypogaea*

**Figure 7:**
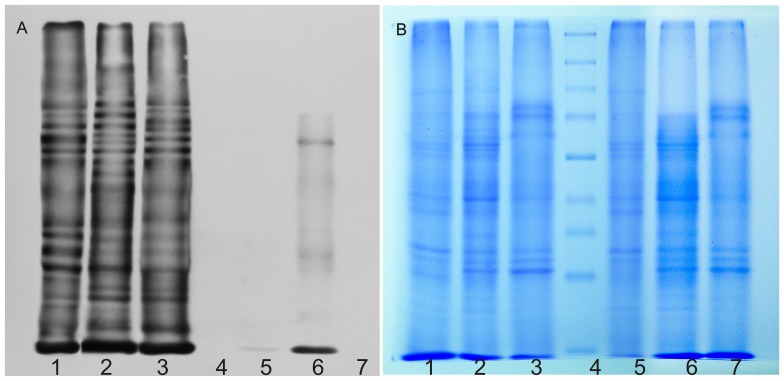
Detection of S-nitrosylated proteins in *A. hypogaea* nodules. Bacteroid free nodule extracts of 20, 40 and 80 day were processed as per described in the S-nitrosylated protein detection assay kit of Cayman Chemicals, Ann Arbor, Michigan with slight modification. Gel profile of the blot is representative of results obtained from the analysis of three independent experiments. (A) Lane 1, 2 and 3 depict reduced and biotinylated samples of 20, 40 and 80 day old nodule extracts. Lane 4 represents the marker. Lane 5, 6 and 7 represent the endogenously biotinylated protein profiles correspond to lanes 1, 2 and 3. (B) is the corresponding gel profile of blot (A).

S-nitrosylation is emerging as a key redox based post translational modification during plant immune function. The modulation of cellular SNO level as well as S-nitrosylation was found to regulate the accumulation of the plant immune activator, salicylic acid (SA) and expression of SA-dependent genes by TGA1, the transcriptional regulator [40]. Although there is increasing evidence of finding nitrosothiols in several plant samples [41], [42], the presence of nitrosothiols in *A. hypogaea* nodules is still novel information in legumes. Figure 7 represents S-nitrosylated protein profile of *A. hypogaea* nodule extracts. Lane 1, 2 and 3 represent reduced and biotinylated samples of 20, 40 and 80 day old nodule extracts. Lane 4 represents the marker. Lane 5, 6 and 7 represent the endogenously biotinylated protein profiles corresponding to lanes 1, 2 and 3. In the bacteroid free nodule extract, mixture of proteins with free cysteine thiols and proteins with cysteine S-nitrosylation could be present. So first free cysteine thiols were blocked by *S*-methylthiolation with MMTS (methyl methanethiosulfonate); then S-nitrosylated cysteines or SNOs were converted to thiols via transnitrosation with ascorbate; then the nascent thiols were labeled in situ by S*-*biotinylation with N-[6-(Biotinamido)hexyl]-3′-(2′-pyridyldithio)propionamide (biotin-HPDP), a reactive mixed disulfide of biotin. So test of endogenously biotinylated protein samples with the probe is essential to detect the actual profile of S-nitrosylated proteins in the sample. Endogenously biotinylated proteins probably vary with the age of the plant. That is why only 40 day old sample represents hybridization with the probe. In other samples the level of endogenously biotinylated proteins could not be detected under our experimental conditions indicating the level of endogenously biotinylated proteins were low. We also checked for protein tyrosine nitration in nodule extracts. We did not observe any significant level of nitrated proteins in nodules of *A. hypogaea* (Figure S3).

### Presence of Low Thiols and Redox Active Enzymes in Functioning Nodules of *A. hypogaea*

In order to gain a precise view of the redox environment of *A. hypogaea* functional nodules, we determined the total thiol content in *A. hypogaea* nodule. The total thiol content was very low in comparison to the total thiol contents in nodules reported in other plants which encourage infection mode of entry [43], [44]. We also measured the glutathione reductase, catalase, ascorbate peroxidase (APx) (EC 1.11.1.11) and superoxide dismutase (SOD) activity in 20, 40 and 80 day old *A. hypogaea* nodule extracts (Table 1). In our system we could only measure the GR activity in 20 and 40 day old nodule extracts. GR activity was found to have increased significantly in 40 day old nodules (8.3±0.49 n mole min^−1 ^mg^−1^) compared with the 20 day old nodules (1±0.15 n mole min^−1 ^mg^−1^). In 80 day old nodule extracts, the expression level of GR was probably very low which could not be detected by our assay system. APx activity did not change much in 20, 40 and 80 day old nodules ranging from 0.38±0.007 to 0.5±0.03 µ mole min^−1 ^mg^−1^ proteins. Interestingly, the specific activity of SOD was found to be high in all three time points ranging from 90.53±5.38 to 133.23±9.66 µ mole^−1 ^min^−1 ^mg^−1^ compared to the reported SOD activity (49 µ mole min^−1 ^mg^−1^) of soybean nodules [45]. On the other hand, the catalase activity was found to be lower in 20, 40 and 80 day old nodules (sp. activity ranging from 12.58±0.18 to 17.35±1.36 µ mole min^−1 ^mg^−1^) compared to the specific activity of the crude soybean nodule extracts (172 µ mole min^−1 ^mg^−1^) of 30 day after their appearance in the roots [45]. Next we measured the Glutathione peroxidase (GPx) (EC 1.11.1.9) and S- nitrosoglutathione (GSNO) reductase (EC 1.1.1.284) enzyme activities in nodule extracts. But GPx and GSNO reductase activities could not be detected in *A. hypogaea* nodule extracts. GSNO reductase activity was reported only in *Arabidopsis thaliana* [46] and pea leaves [47].

**Table 1:** Measurement of total thiol, Glutathione reductase (GR), Catalase, Ascorbate Peroxidase and Superoxide dismutase (SOD) activity in different days’ nodule extracts of *A. hypogaea*.

Parameters	20 day Nodule	40 day Nodule	80 day Nodule
Total thiol (n mole mg^−1^protein)	1.27±0.02	1.37±0.04	1.39±0.10
GR (n mole min^−1^ mg^−1^)	1.08±0.15	8.30±0.49	ND
Catalase (µmole min^−1 ^mg^−1^)	17.35±1.36	12.58±0.18	12.55±0.85
Ascorbate Peroxidase (µ mole min^−1 ^mg^−1^)	0.388±0.007	0.50±0.03	0.498±0.04
SOD (µmole min^−1 ^mg^−1^)	133.23±9.66	90.53±5.38	107.62±7.99

Total thiol content was measured in 20, 40 and 80 day nodule extracts as described by Akerboom and Sies, (1981). Glutathione Reductase activity was measured spectrophotometrically at 340 nm over 2 minute by following NADPH oxidation whereas Catalase and Ascorbate Peroxidase activity was measured by following the H_2_O_2_ oxidation spectrophotometrically at 240 nm for 2 min in 20, 40, 80 day nodule extracts of *A. hypogaea.* SOD activity was measured in the same samples using xanthine oxidase and NBT and absorption was measured at 560 nm. Results are expressed as mean ± SD, for n = 3 experiments. P≤0.01, using one-way ANOVA.

### Bacterial Redox Active Enzymes and GSNO Reductase Protect them Against Nitrosative Stress

**Figure 8:**
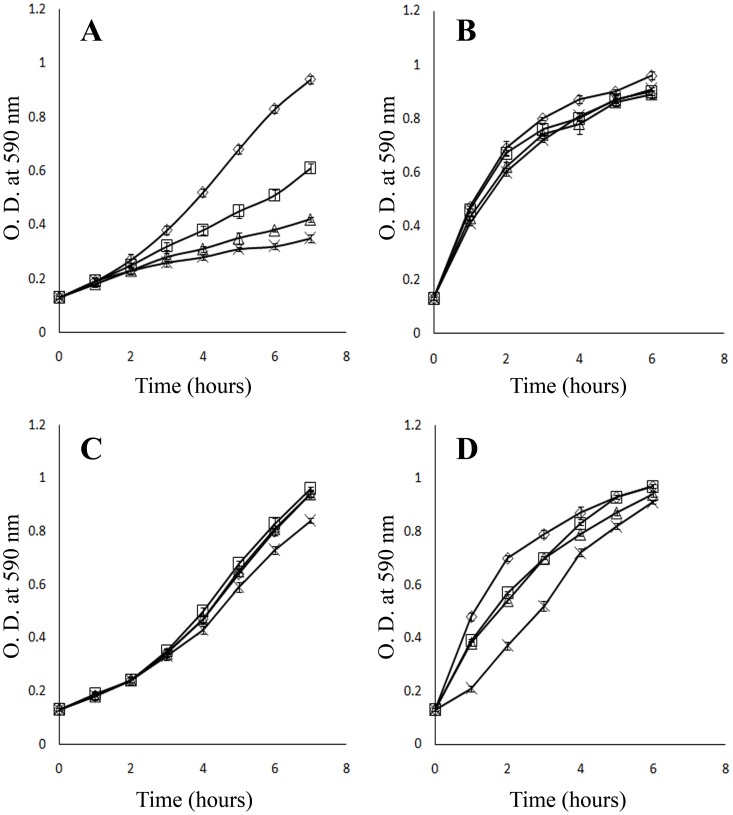
Effect of sodium nitroprusside (SNP) and GSNO on the growth of *S. meliloti* and *Bradyrhizobium sp*. Growth curves of *S. meliloti* (A) and *Bradyrhizobium sp* (B) in the presence of different concentrations of SNP: Mid-log phase cells (OD_590_ = 0.5–0.7) were diluted in fresh medium containing graded concentration of SNP (—◊— control, —□— 0.5 mM, —Δ— 1.0 mM, and —×— 3.0 mM) and were grown under their respective growth conditions and media. Same procedures were performed to study the effect of GSNO (—◊— control, —□— 0.5 mM, —Δ— 1.0 mM, and —×— 3.0 mM) on growth of *S. meliloti* (C) and *Bradyrhizobium sp* (D).

Our microscopic data clearly showed that bacteroids were exposed to nitrosothiols. So we wanted to check the response of the symbiont against the reactive nitrogen species. For this we used sodium nitroprusside (SNP) and S-nitrosoglutathione (GSNO) as the NO donating compound to create nitrosative stress on these bacteria. We tested the capacity of GSNO and SNP to inhibit the growth of these two organisms in aerobic condition. Figure 8 represents the growth curves of *S. meliloti* 1021 and *Bradyrhizobium sp* in the presence of different concentrations of GSNO and SNP. There was no significant growth inhibition up to 1 mM GSNO in *S. meliloti* 1021. The growth of *S. meliloti* 1021 was clearly affected at 0.5 mM, 1 mM and 3 mM SNP. The growth of *Bradyrhizobium sp* was not significantly affected in the presence of SNP. However, *Bradyrhizobium* cells were sensitive to GSNO. There was a clear lag period in the growth curve in presence of 3 mM GSNO concentrations. However, cells were capable to reach an absorbance value close to the control set after six hours.

In eukaryotes and prokaryotes, the tripeptide glutathione (GSH) in its reduced state acts as an important constituent of cellular first line of defense against oxidative stress and helps to maintain intracellular redox balance. So we investigated the effect of nitrosative stress on cellular glutathione level both in *Bradyrhizobium sp* and *S. meliloti* (Table 2). We measured total glutathione (GSH + GSSG), oxidized glutathione (GSSG) and calculated reduced glutathione (GSH) as markers of cellular glutathione status following 1 mM and 3 mM SNP stress for 30 min. There was a ∼ 3.3 fold increase in total thiol in *S. meliloti* 1021 following 3 mM SNP treatment compared to the control set. However, in *Bradyrhizobium sp* fold increase in total thiol was much less i.e. ∼1.76 following 1 mM SNP exposure under similar experimental conditions. The basal level total thiol GSH was higher (∼ 5.84 fold) in *S. meliloti* than *Bradyrhizobium sp.* We could not detect the GSSG and GSH level in control and 1 mM SNP treated *Bradyrhizobium sp* under our experimental conditions [48]. However, detectable amount of GSSG and GSH were found following 3 mM SNP exposure for 30 min in *Bradyrhizobium sp.* There was a two fold increase in GSSG content following 3 mM SNP treatment in *S. meliloti.* But it did not affect the cellular reducing environment as there was a 3.36 fold increase in GSH level in *S. meliloti* following 3 mM SNP treatment for 30 min which resulted in 1.76 fold increase in GSH/GSSG ratio.

**Table 2:** Quantification of thiol pool in *S. meliloti* 1021 and *Bradyrhizobium sp* after 30 minute stress with SNP in media.

	*S. meliloti*			*Bradyrhizobium sp*		
Parameters	Control	1 mM SNP	3 mM SNP	Control	1 mM SNP	3 mM SNP
Total thiol (nmole mg^−1^protein)	5.03±0.19	4.8±0.14	16.46±0.06	0.86±0.06	1.52±0.08	1.39±0.04
Oxidized Glutathione (nmole mg^−1^protein)	0.32±0.006	0.13±0.006	0.61±0.04	ND	ND	0.14±0.01
Reduced Glutathione (nmole mg^−1^protein)	4.71±0.19	4.31±0.006	15.85±0.11	ND	ND	1.24±0.05
Ratio of Reduced to Oxidized Glutathione	14.72±0.53	33.15±1.41	25.98±2.02	ND	ND	8.86±0.98

*S. meliloti* cells were grown to mid log phase at 30°C in TY media whereas *Bradyrhizobium sp* cells were grown similarly in YEM media. Sodium nitroprusside (SNP) was added to mid log phase cell in the final concentration of 1.0 and 3.0 mM and incubated at 30°C for 30 minute. Cells were harvested, lysed and cell free crude extracts were subjected to quantification of total thiol, oxidized glutathione, reduced glutathione and ratio for reduced to oxidized glutathione. Results are expressed as mean ± SD, for n = 3 experiments. P≤0.01, using one-way ANOVA.

To investigate the cellular capacity to restore the GSH content after nitrosative stress, GR and GPx activities were assessed using 1 mM and 3 mM SNP treatment under similar experimental conditions. Interestingly, basal level GR was much higher (∼ 3 fold) in *Bradyrhizobium sp* compared to *S. meliloti* (Table 3) which indicated that cells are well equipped for recycling of GSSG, generated under oxidative or nitrosative stress. There was a little change in GR in *Bradyrhizobium sp* following 1 mM and 3 mM SNP exposure.

**Table 3:** Assessment of Glutathione reductase (GR), Catalase, Glutathione peroxidase (GPx) and GSNO reductase activity in *S. meliloti* 1021 and *Bradyrhizobium sp* after 30 minute stress with SNP in media.

	*S. meliloti*			*Bradyrhizobium sp*		
Parameters	Control	1 mM SNP	3 mM SNP	Control	1 mM SNP	3 mM SNP
GR (nmole min^−1^ mg^−1^)	54.5±2.86	49.96±0.72	53.92±1.41	161.98±2.48	185.45±2.59	203.14±7.66
Catalase (µmole min^−1^ mg^−1^)	9.01±0.17	9.55±0.75	9.83±0.47	121.88±0.93	132.62±8.38	112.02±9.50
GPx (nmole min^−1^ mg^−1^)	1.1±0.06	1.63±0.10	1.62±0.08	3.84±0.16	4.04±0.25	4.46±0.20
GSNO Reductase (nmole min^−1^ mg^−1^)	79.3±1.00	89.78±4.18	96.06±3.06	76.81±5.50	89.6±3.41	95.37±2.95

*S. meliloti* cells were grown to mid log phase at 30°C in TY media whereas *Bradyrhizobium sp* cells were grown similarly in YEM media. Sodium nitroprusside (SNP) was added to mid log phase cell in the final concentration of 1.0 and 3.0 mM and incubated at 30°C for 30 minute. Cells were harvested, lysed and cell free crude extracts were subjected to assessment of GR, Catalase, GPx and GSNO reductase activity. Results are expressed as mean ± SD, for n = 3 experiments. P≤0.01, using one-way ANOVA.

The basal level catalase of *Bradyrhizobium sp* was 13.5 fold higher than *S. meliloti*. However, there was no significant change in catalase level in *Bradyrhizobium sp* following SNP treatment. Similar result was obtained in *S. meliloti*. Interestingly GPx level was also high in *Bradyrhizobium sp* compared to *S. meliloti* (Table 3). Both *Bradyrhizobium sp* and *S. meliloti* possess GSNO reductase activity. The basal level GSNO reductase activity was almost same in both the organisms but it was induced (∼ 24% increase) in presence of 3 mM SNP in both the organisms.

## Discussion

Symbiotic interactions between legumes and rhizobia represent an original physiological context that results from a combination of developmental and infectious events. The importance of NO during the interaction of plants with pathogens brought us to characterize the occurrence of this molecule during the infection of the model legume *A. hypogaea* by its bacterial partner *Bradyrhizobium sp*. There is little information available on the crack entry legumes regarding the nitric oxide production and its effect on the partner microorganisms [49]. In this study, we used a biochemical approach to characterize the nodule environment of *A. hypogaea* in terms of reactive nitrogen species and the effect of nitrosative stress on the symbiont. Furthermore, we first report the presence of nitrosothiols and nitrosylated proteins in nodules of *A. hypogaea.* It is evident from our fluorimetric as well as biochemical study that RSNOs are present in a substantial amount in *A. hypogaea* nodules. It has been reported that RSNOs formed from low molecular weight biological thiols (L-cysteine and glutathione) are degraded in the presence of superoxide [50] producing nitrite and nitrate in the reaction mixture. ROS detoxifying enzymes like SOD, APx and catalase were found to become high in *A. hypogaea* nodule environment. That is why we did not observe any superoxide in the nodule environment. Absence of ROS and nitric oxide as determined by fluorescent probes distinguished *A. hypogaea* nodule as a separate group of plant where the signaling molecules related to the symbiotic association are different. There are multiple pathways of NO production thought to exist in plant [7], [51]. These route(s) may control NO production but the bioavailability of NO depends on its turnover. NO can be metabolized to nitrate by nonsymbiotic hemoglobins. NO also reacts with GSH to form GSNO, which can release NO or functions as a transnitrosylating agent and can be degraded by GSNO reductase. NO can also react with superoxide to form peroxynitrite. So a network of RNS transformation and turnover balance the bioavailability of these signal compounds.

Flavohemoglobins (Hmp) are proteins involved in NO detoxification in many bacterial species. In *S. meliloti*, the role of Hmp protein has been demonstrated by the team through the production of hmp-null mutants and hmp-overexpressing (*hmp++*) strains [49]. RSNOs can be decomposed by various pathways that may include thiol [52], glutathione peroxidase [53] and GSNO reductase [54], [55]. GSNO is equilibrated with other RSNO through transnitrosation. As a result GSNO reductase can indirectly regulate the concentration of RSNOs in vivo. However, GSNO reductase is absent in *A. hypogaea* nodule extract. In our growth curve study we found that both *S. meliloti* and *Bradyrhizobium sp* are affected in presence of the graded concentrations of GSNO. Interestingly, both *Bradyrhizobium sp and S. meliloti* were found to contain substantial level of GSNO reductase activity. So the presence of GSNO reductase activity in bacteria might reduce the actual concentrations of GSNO in which they were exposed. So the bacterial GSNO reductase might act as potential defense enzyme along with other redox reactive enzymes like GR, GPx, and catalase against RSNOs in *A. hypogaea* nodules.

**Figure 9:**
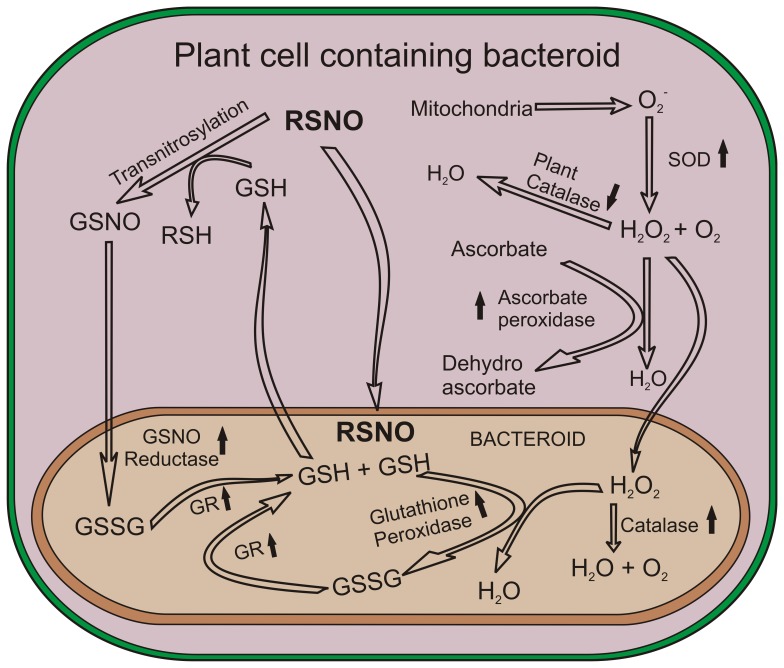
Representation of maintenance of Redox Cycle in nodules and bacteroid compartments. Scheme represents the nodule redox environment, and transport of redox molecules in plant cell compartment and bacteroid compartment. Based on the biochemical analysis of thiols, RSNO and redox active enzymes of plant cell and rhizobia, the exchange of molecules between the compartments has been shown. Upward arrow head indicates upregulation, downward arrowhead indicates downregulation. RSNO could be produced in the nodule compartment by several routes. Reduced glutathiones (GSH) take part in transnitrosylation reaction in nodule compartment. GSNO produced in nodule compartment may diffuse to the bacteroid cell where GSNO reductase could convert it to GSSG and NH_3_; GSSG is recycled by GR and GPx. High basal level GR present in the bacteroid could recycle GSSG to GSH. GSH from the bacteroid may be transported to the nodule compartment to take part in the trans nitrosylation reaction. H_2_O_2_ produced in nodule compartment could be detoxified by nodule catalase, and APx and the excess H_2_O_2_ might be diffused to the bacteroid compartment where *Bradyrhizobium* could detoxify it with high level of catalase and GPx.

In the biological system, S-nitrosylated proteins can be formed through a number of mechanisms that include transnitrosylation, metalloprotein catalyzed reactions, or reaction with electrophilic nitrogen oxide species. But the actual mechanisms of S-nitrosylation as well as their functions in *A. hypogaea* nodules are not known. Together our data indicate that nodule environment of crack entry legumes is different than the nodules of infection mode entry in terms of NO, ROS and RN*S.* Based on our biochemical characterization, we propose a scheme (Figure 9) for the exchange of redox molecules and reactive chemical species between the bacteroid and nodule compartment. In future, the identification of nitrosylated proteins could provide new insights into the redox based machinery that controls the nodule environment as well as effective symbiosis in *A. hypogaea* nodule.

## Materials and Methods

### Chemicals

All the reagents are of highest purity and purchased from Sigma Chemical Co., (St. Louis, MO USA) unless otherwise stated.

### Plant Material

*A. hypogaea* JL 24 (ICG 7827) was a generous gift from ICRISAT, Patancheru 502 324, Andhra Pradesh and were cultivated in the village fields of Gopalnagar, P.O. – Malighati, Paschim Medinipur, West Bengal, state of India as seasonal crop. No specific permits were required for the described field studies. The location is not protected in any way and it is confirmed that the field studies did not involve endangered or protected species. Nodules were collected from the roots of 22, 40 and 80 day old plantations. *M. sativa* nodules of 143 day were collected from field.

### Bacterial Strains, Media and Growth Conditions

*Bradyrhizobium sp* NC 921001 is the partner organism of *A. hypogaea* JL 24 and *S. meliloti* 1021 is the partner organism of *M. truncatula* and *M. sativa. Bradyrhizobium sp* NC 921001 was a generous gift from Dr. Maitrayee Das Gupta, Dept. of Biochemistry, C.U. and *S. meliloti* 1021 was kindly donated by Elena Gartner, of Australian National University. *S. meliloti* cells were grown at 30°C in complex tryptone yeast extract (TY) medium (tryptone 0.5% (w/v), yeast extract 0.3% (w/v), ) containing 10 mM CaCl_2_ and supplemented with streptomycin 100 µg ml^−1^. *Bradyrhizobium sp* NC 921001 was grown at 30°C in complex yeast mannitol broth (YEM) of HIMEDIA.

### Specific Identification of Reactive Oxygen Species (ROS) and Nitric Oxide (NO) by Fluorescence Microscopy

ROS were detected using the fluorescent reagent Dichlorofluorescein diacetate (DCF-DA) and NO was detected with 4, 5-diaminofluorescein diacetate (DAF-2DA, Calbiochem). *A. hypogaea* and *M. sativa* nodule sections were incubated with 10 µM DCF-DA as well as 10 µM DAF-2DA at 25°C for 1 h in darkness, and then washed thrice. Nitric oxide in *M. sativa* nodules were quenched by incubating the nodule sections in 1 mM cPTIO for 1 hour in darkness before incubation with DAF-2DA. The images of the sections were captured using green fluorescence (excitation 485 nm; emission 530 nm) for DCF-DA and green fluorescence (excitation 495 nm; emission 515 nm) for DAF-2DA.

### Detection of Thiols and/or Nitrosothiols by Fluorescence Microscopy

Thiols and/or nitrosothiols were detected using the fluorescent reagent Alexa fluor 488 Hg-link phenylmercury [42]. *A. hypogaea* as well as *M. sativa* nodule sections were incubated with 10 µM Alexa fluor 488 Hg-link phenylmercury (Molecular Probes) at 25°C for 2 h in darkness washed thrice and images were captured with fluorescence microscope using green fluorescence (excitation 495 nm; emission 519 nm).

### Blocking of Nitrosothiol

Three sets were prepared for the experimental study. In one, the nodule cross sections were treated with 50 mM N-ethyl-maleimide (NEM) prepared in ethanol and was incubated at 25°C for 2 h in darkness. In the other, the nodule cross sections were incubated with 20 mM ascorbate prepared in HEN buffer for one hour at room temperature followed by similar NEM treatment. The last set prepared as control was only treated with buffer without ascorbate and NEM. Then all the sections were stained with 10 µM Alexa fluor 488 Hg-link phenylmercury (Molecular Probes) and images were captured as stated previously.

### Bacteroid Detection

Bacteroids were detected together with RSNO in fresh nodule slices. The nucleic acid dye Syto83 (Invitrogen) was added with alexafluor 488 both at a final concentration of 10 µM. The sections were incubated for 1 hour followed by three washings. Syto83 emission was recorded using an upper 560 band-pass filter upon excitation at 543 nm with a helium laser. The bacteroids of *M. sativa* were stained only with Alexa fluor 488. The emission of Alexa fluor 488 Hg-link phenylmercury was recorded using 505 to 570 band-pass filter upon excitation at 495 nm.

### Preparation of Bacteroid Free Nodule Cytosolic Extract

Bacteroid free nodule extracts were prepared as stated by Oehrle et al. (2008) [56] with slight modifications. Nodules were removed from the roots and were immediately placed on ice. 1 g of nodules were homogenized in a pre-chilled mortar and pestle with 2 ml of pre-chilled extraction buffer that consists of 100 mM Tris-HCl of pH 8.8 with 17% (w/v) sucrose, 100 mM DTT, 1 mM PMSF, 10 mM EDTA, 20 µg ml^−1^ pepstatin A, 20 µg ml^−1^ leupeptin and 20 µg ml^−1^ aprotenin. To precipitate the plant phenolic compounds, 0.33 g of insoluble Poly(N-vinyl -2-pyrrolidone) (PVP) was used for per g of nodule. Homogenized nodules were filtered by passing through four layers of cheese-clothes those were pre-moistened with extraction buffer. Next the filtrates were centrifuged at 400 g for 10 minute. The supernatants containing the soluble plant proteins and bacteroids were centrifuged at 8,000 g for 20 min to pellet the bacteroids and the clear supernatant was used for enzymatic assay and S-nitrosylation determination.

### Preparation of Bacterial Cell Free Extract

*Bradyrhizobium sp* and *S. meliloti* 1021 were grown up to mid log phase at 30°C. Sodium nitroprusside (SNP) was added to mid log phase cell in the final concentration of 1.0 and 3.0 mM and was incubated at 30°C for 30 minute. Next cells were pelleted down by centrifugation at 3,400 g for 5 minute at 4°C. Cell pellets were washed thrice with pre chilled phosphate buffer saline (PBS) (NaCl 0.8% w/v, KCl 0.02% w/v, Na_2_HPO_4_ 0.144% w/v, and KH_2_PO_4_ 0.024% w/v, pH 7.0). Pellets were resuspended in minimal volume of PBS containing 1 mM PMSF, 1 mM EDTA, 20 µg ml^−1^ pepstatin A, 20 µg ml^−1^ leupeptin and 20 µg ml^−1^ aprotenin just to make slurry and was left in ice for 15 minute. Lysozyme solution was added to the slurry at a final concentration of 1 mg ml^−1^ and again was kept in ice for 15 minute. Then the cells were sonicated using 6 pulses each for 10 seconds at each 10 second intervals at highest amplitude percentage of in Hilscher UP50H sonicator. Total procedure was performed on ice to prevent the temperature increase and was centrifuged at 13,000 g for 15 minute at 4°C. The supernatants were collected and were used for enzyme assay.

### Biochemical Measurement of S-Nitrosothiol Content with the Saville Assay

The S-nitrosothiol content was determined by the Saville method [57]. For the analysis of the S-nitrosothiol content, three sets of solutions were prepared. Solution A consisted of 1% sulfanilamide dissolved in 0.5 M HCl, solution B contained 1% sulfanilamide and 0.2% HgCl_2_ in 0.5 M HCl and solution C was prepared by dissolving 0.02% N-(1-naphthyl) - ethylendiaminedihydrochloride in 0.5 M HCl. Two protein samples were prepared in 100 mM phosphate buffer (500 µl). Solution A (250 µl) was then added to one of the samples, and the same amount of solution B was added to the other. After 5 minute, when the formation of the diazonium salt was complete, 250 µl of solution C were added to each of the two samples. After 5 minute, color formation of the azo dye was complete and the absorbance of 540 nm was recorded spectrophotometrically. The absorbance at 540 nm of the reaction with solution A was for the presence of free nitrite in plant samples and the absorbance at the same wavelength with solution B was for the presence of free nitrite and nitrosothiol in plant samples. So the actual nitrosothiol content in plant sample was the difference in absorbance between solution A and solution B. Finally the nitrosothiol content was determined in terms of nitrite from the standard curve of nitrite.

### Preparation of GSNO

Preparation of GSNO solution was performed according to Hart (1985) [58] with slight modifications. 1 M solution of NaNO_2_ was prepared in distilled water and 1 M solution of GSH was prepared in 1 N HCl. These two solutions were mixed on ice (1∶1, v/v) to obtain GSNO solution and GSNO concentration was determined using ε_335_ nm = 586 M ^−1^ cm^−1^.

### Bacterial Growth Study in Presence of SNP and GSNO

Mid log phase cultures of bacterial cells were used as inoculum following dilution in fresh media to O.D._590_ = 0.13–0.15 in the presence of graded concentrations of SNP or GSNO. Cell growth was monitored by measuring the cell density spectrophotometrically at 590 nm. SNP and GSNO were kept in 0.1 M Tris buffer, pH-7.4 at room temperature for three days in presence of light for decomposition. In all the above experiments, control sets were performed using decomposed NO donors.

### Assessment of Enzymatic Status

#### Glutathione reductase and catalase

Glutathione reductase (GR) and catalase activities were measured in the cytosolic fractions of plant nodules, *Bradyrhizobium sp* and *S. meliloti*. According to Carlberg et al. (1975) [59] NADPH oxidation was followed spectrophotometrically at 340 nm over 2 min to assay Glutathione Reductase. Catalase activity was measured according to Aebi (1984) [60] following the H_2_O_2_ degradation spectrophotometrically at 240 nm over 2 minute.

#### GSNO reductase

GSNO reductase activity of crude cell-free extracts were determined according to Liu et al. (2001) [54] by measuring the decrease in absorbance at 340 nm due to utilization of NADH. The specific activity was calculated using the extinction coefficient of NADH (6.22 mM^−1 ^cm^−1^) and was expressed in n mol min^−1 ^mg^−1^ protein.

#### Glutathione peroxidase and ascorbate peroxidase

The Glutathione peroxidase (GPx) activity of crude cell-free extracts were determined according to Wendel (1981) [61] by measuring the change in absorbance at 340 nm due to utilization of NADPH. Ascorbate peroxidase (APx) assay was performed according to Miyake et al. (1992) [62]. The H_2_O_2_ dependent oxidation of ascorbate was followed by monitoring the decrease in absorbance at 240 nm and the amount of consumed ascorbate was determined by using absorption coefficient 2.8 mM^−1 ^cm^−1^. One unit of APx was defined as the enzyme that oxidized 1 µ mol of ascorbate per minute at 25°C.

#### Superoxide dismutase

The superoxide dismutase (SOD) assay was performed using xanthine oxidase (XO)/NBT system according to Ukeda *et al.,* (1997) [63] with slight modification. The reaction was performed in 0.5 ml of 50 mM potassium phosphate buffer that consisted of 0.125 mM xanthine, 0.125 mM EDTA, 0.03 mM NBT and SOD solution. The reaction was initiated by the addition of XO. The change of absorbance was monitored at 560 nm at 25°C for 2 minute. One unit of SOD was defined as the amount of enzyme that caused a 50% decrease in SOD - inhibited NBT reduction.

### Total Glutathione (GSH + GSSG), Reduced Glutathione (GSH) and Oxidized Glutathione (GSSG) Content Measurement

The measurements of these three parameters were performed according to the method described by Akerboom and Sies, (1981) [48]. The crude cell free extracts were first deproteinised and were centrifuged at 5,000 g for 5 minute. Then cell free extracts were neutralized with KOH containing 0.3 M HEPES. These neutralized samples were then centrifuged at 5,000 g for 5 minute and were utilized for determination of the above mentioned parameters. Total glutathione (GSH+2GSSG) concentrations were estimated using glutathione reductase dependent DTNB reduction method. The reactions were performed in 100 mM potassium phosphate buffer of pH 7.0 containing 1 mM EDTA, 0.12 U GR, 0.2 mM NADPH, 0.063 mM DTNB with a total volume of 500 µl. The same neutralized samples were used for GSSG estimation after addition of 2-vinyl pyridine (Lancaster Chemicals, England) (50∶1, v/v) and were incubated for 1 hour. The contents of reduced glutathione were determined from the difference between total and oxidized glutathione contents of the samples and both GSH and GSSG concentration were expressed in n mol mg^−1^ protein.

### Determination of Protein S-nitrosylation

The detection of protein S-nitrosylation was based upon the modified Biotin switch technique described by Jaffrey et al. (2001) [64]. Each 400 µg proteins from bacteroid free nodule extracts of 20, 40 and 80 day were taken and were processed according to the method described in the S-nitrosylated protein detection assay kit of Cayman Chemicals, Ann Arbor, Michigan. 5 µg proteins of each processed sample were mixed with Laemmli buffer and were incubated in a boiling water bath for 5 min followed by 5 min on ice prior to loading to covalently bind the biotin label. Next the samples were separated in 10% SDS-polyacrylamide gel electrophoresis. Then the proteins were blotted on PVDF membrane. The membrane was blocked at 4°C for overnight. Next day the blot was washed with TBS for twice and incubated with 1∶200 dilution of S-nitrosylation Detection Reagent I (HRP) in TBS for 1 hour at room temperature. Then the membrane was washed extensively with TBS and immunopositive bands were visualized by using chemilluminiscent reagent (Pierce ECL Western Blotting Substrate) as directed by the manufacturer.

### Detection of Bacteroid Free Nodule Protein Nitration

The proteins of nodule cytosolic extracts were separated with 10% SDS PAGE. Next the proteins were blotted on PVDF membrane and blocked overnight at 4°C. PVDF membrane containing proteins was then incubated for 60 min at room temperature with anti nitrotyrosine monoclonal antibody (Upstate, USA) at 1∶2000 dilutions. Next the membrane was washed and was incubated with a HRP conjugated goat antimouse IgG antibody (Santa Cruz biotechnology) at 1∶500 dilutions. After that membrane was washed extensively and the immunopositive spots were visualized by using chemilluminiscent reagent (Perkin Elmer, Boston, MA) as directed by the manufacturer.

## Supporting Information

Figure S1 **Detection of NO in nodule sections of** ***M. sativa*** **using DAF-2DA with C-PTIO.** Nodule sections (A, C, E, and G) were first incubated with 1 mM cPTIO for 1 hour in darkness. Next the nodule sections were incubated with 10 µM DAF-2DA at 25°C for 1 hour in darkness. Photographs are representative of results obtained from the analysis of nodules in three independent experiments. Images of *A. hypogaea* nodule sections showed absence of NO dependent DAF-2DA fluorescence (green colour) in (A) 20 day, (C) 40 day and (E) 80 day old nodules. In image (G) 143 day old *M. sativa* nodule section also showed the absence of NO dependent DAF-2DA fluorescence (green colour). (B), (D), (F) and (H) showed the corresponding bright field images. Background fluorescence of 143 day old *M. sativa* nodule section was shown in image (I). (J) is the corresponding bright field image. Scale bar = 100 µm.
(TIF)

Figure S2 **ROS detection in** ***A. hypogaea*** **JL 24 and** ***M. sativa*** **nodules with DCFDA.** All the nodule sections were incubated with 10 µM DCF-DA at 25°C for 1 hour in darkness. Photographs are representative of results obtained from the analysis of nodules in three independent experiments. Images of nodules showed absence of ROS dependent DCFDA fluorescence (green colour) in (A) 20 day, (C) 40 day and (E) 80 day old nodules of *A. hypogaea* and (G) 143 day old *M. sativa* nodule sections. (I) *A. hypogaea* nodule sections were incubated first in presence of H_2_O_2_ and then in presence of DCF-DA at 25°C as positive control (B), (D), (F), (H) and (J) showed the corresponding bright field images. Scale bar for (A), (C) and (I) = 200 µm and for (E) and (G) = 100 µm.
(TIF)

Figure S3 **Protein nitration profile of different day** ***A. hypogaea*** **JL 24 nodule extracts.** Western blots are representative of results obtained from the analysis of nodules in three independent experiments. (A) Protein tyrosine nitration in 20 day (lane 1), 40 day (lane 2) and 80 day (lane 3) old nodule extracts of *A. hypogaea* JL 24. Lane 4 depicts the basal level tyrosine nitration of *S. cerevisiae* as positive control. (B) is the corresponding gray scale coomassie stained gel.
(TIF)
