# Supplementary figures and images for: Detection of S-Nitrosothiol and Nitrosylated Proteins in Arachis hypogaea Functional Nodule: Response of the Nitrogen Fixing Symbiont
Source: PLoS One. 2012 Sep 19;7(9):e45526. doi: 10.1371/journal.pone.0045526 (PMC3446898; doi:10.1371/journal.pone.0045526)

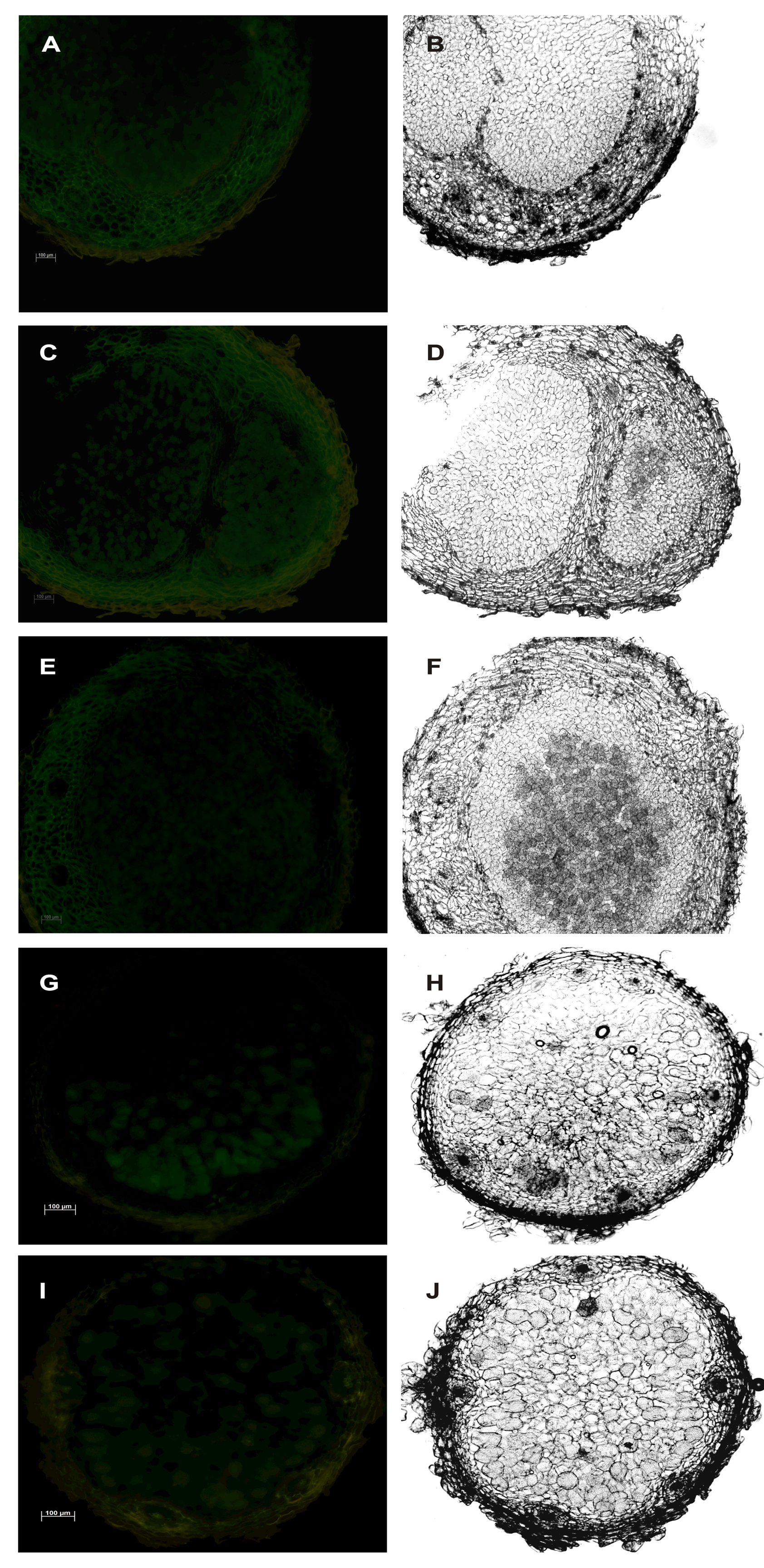

Supplement: Figure S1 — Detection of NO in nodule sections of M. sativa using DAF-2DA with C-PTIO. Nodule sections (A, C, E, and G) were first incubated with 1 mM cPTIO for 1 hour in darkness. Next the nodule sections were incubated with 10 µM DAF-2DA at 25°C for 1 hour in darkness. Photographs are representative of results obtained from the analysis of nodules in three independent experiments. Images of A. hypogaea nodule sections showed absence of NO dependent DAF-2DA fluorescence (green colour) in (A) 20 day, (C) 40 day and (E) 80 day old nodules. In image (G) 143 day old M. sativa nodule section also showed the absence of NO dependent DAF-2DA fluorescence (green colour). (B), (D), (F) and (H) showed the corresponding bright field images. Background fluorescence of 143 day old M. sativa nodule section was shown in image (I). (J) is the corresponding bright field image. Scale bar = 100 µm. (TIF) [file pone.0045526.s001.tif]

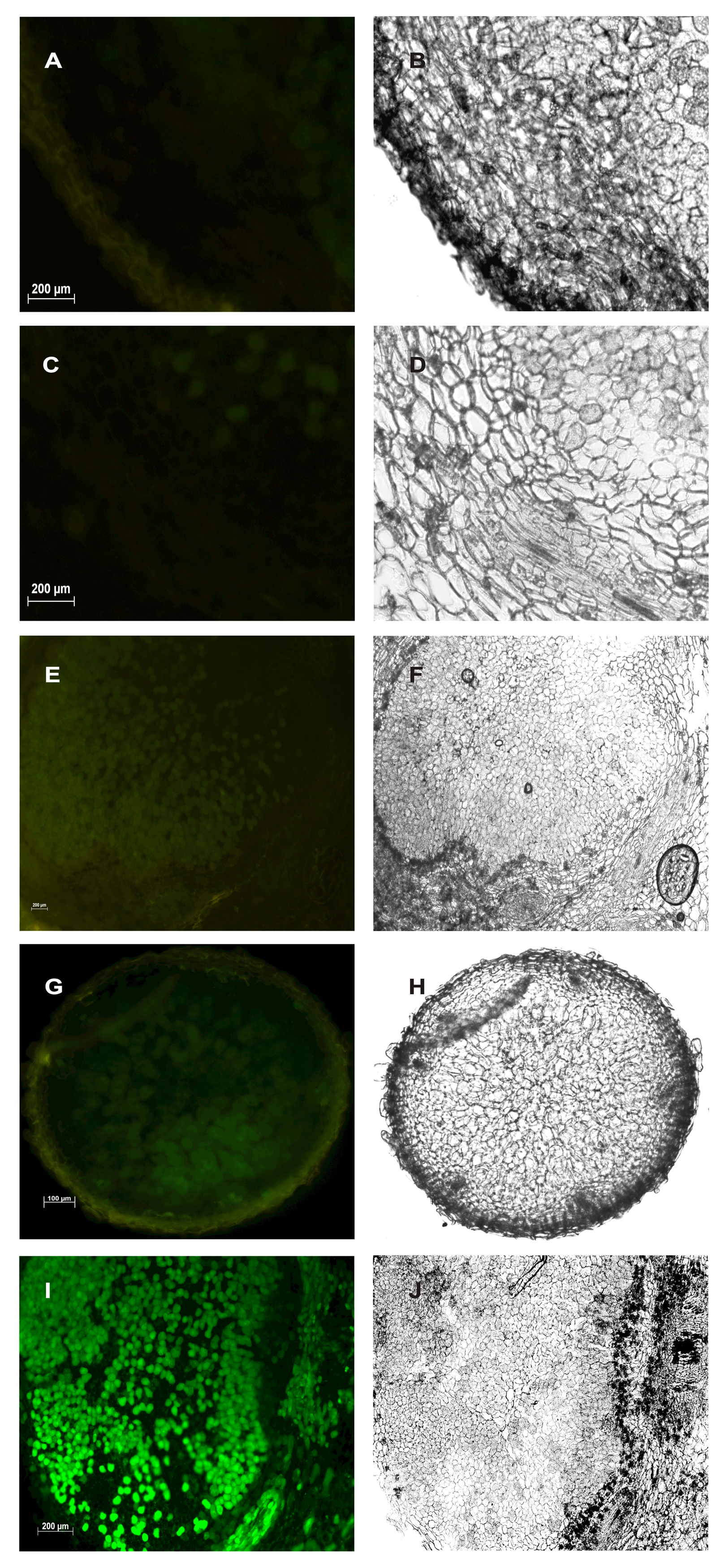

Supplement: Figure S2 — ROS detection in A. hypogaea JL 24 and M. sativa nodules with DCFDA. All the nodule sections were incubated with 10 µM DCF-DA at 25°C for 1 hour in darkness. Photographs are representative of results obtained from the analysis of nodules in three independent experiments. Images of nodules showed absence of ROS dependent DCFDA fluorescence (green colour) in (A) 20 day, (C) 40 day and (E) 80 day old nodules of A. hypogaea and (G) 143 day old M. sativa nodule sections. (I) A. hypogaea nodule sections were incubated first in presence of H2O2 and then in presence of DCF-DA at 25°C as positive control (B), (D), (F), (H) and (J) showed the corresponding bright field images. Scale bar for (A), (C) and (I) = 200 µm and for (E) and (G) = 100 µm. (TIF) [file pone.0045526.s002.tif]

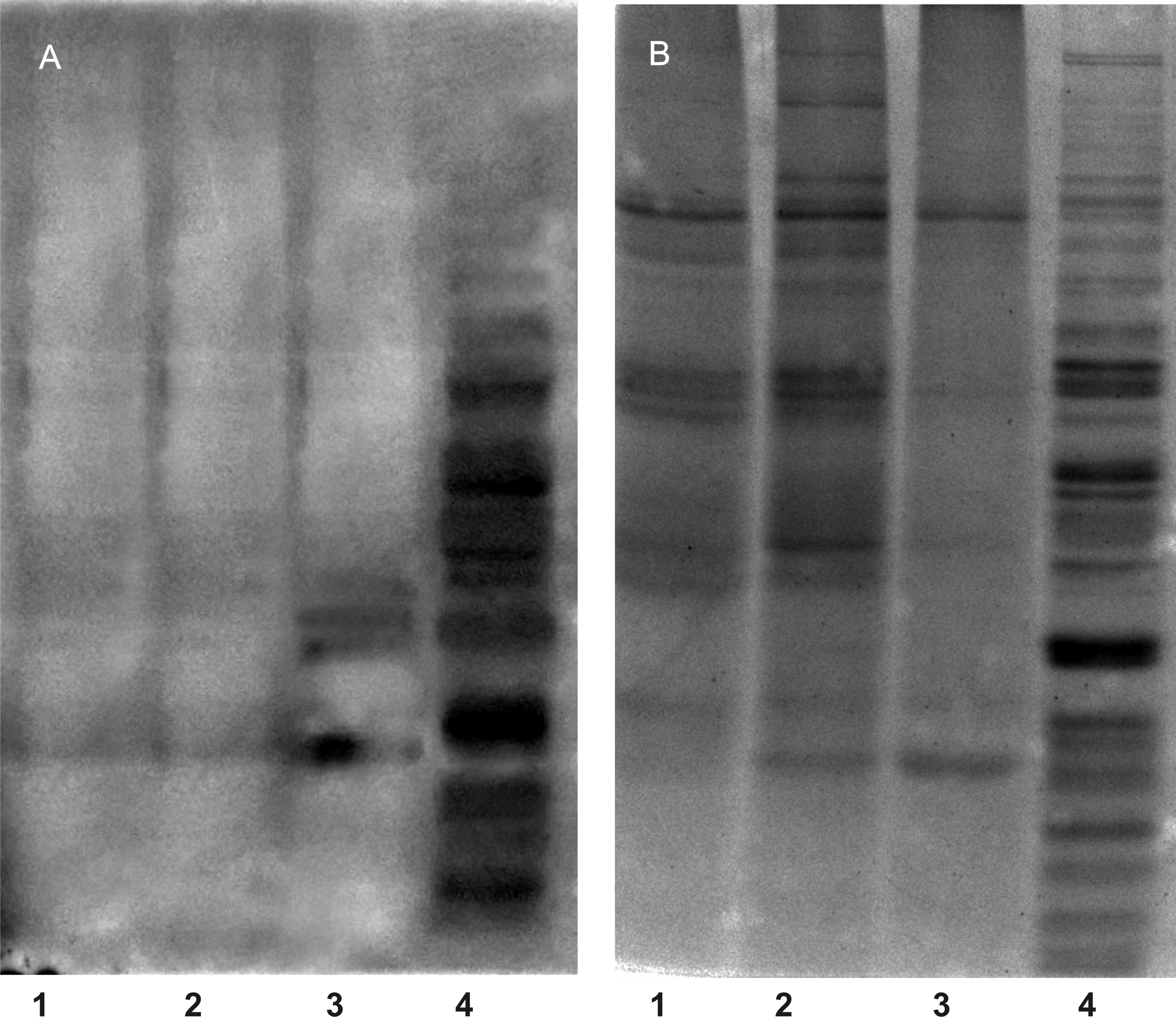

Supplement: Figure S3 — Protein nitration profile of different day A. hypogaea JL 24 nodule extracts. Western blots are representative of results obtained from the analysis of nodules in three independent experiments. (A) Protein tyrosine nitration in 20 day (lane 1), 40 day (lane 2) and 80 day (lane 3) old nodule extracts of A. hypogaea JL 24. Lane 4 depicts the basal level tyrosine nitration of S. cerevisiae as positive control. (B) is the corresponding gray scale coomassie stained gel. (TIF) [file pone.0045526.s003.tif]
